# Supplementary material for: PET-based tracking of CAR T cells and viral gene transfer using a cell surface reporter that binds to lanthanide complexes
Source: Nat Biomed Eng. 2025 Jun 13;9(11):1886–906. doi: 10.1038/s41551-025-01415-7 (PMC12623248; doi:10.1038/s41551-025-01415-7)
Supplement: Supplementary file 1 — Sequences, supplementary figures, discussion, supplementary tables and references. [file 41551_2025_1415_MOESM1_ESM.pdf]

nature biomedical engineering

Supplementary information https://doi.org/10.1038/s41551-025-01415-7

PET-based tracking of CAR T cells and viral gene transfer using a cell surface reporter that binds to lanthanide complexes

In the format provided by the authors and unedited

Content

Sequences 2
Supplementary discussion 1: *In silico* analysis of potential immunogenicity of the DTPA-R 4
Supplementary Table 1 | Prediction of T cell epitopes from DTPA-R by the IEDB.org immunogenicity tool 4
Supplementary Table 2 | Antibodies & staining reagents 6
Supplementary Table 3 | Affinity measurements of CHX-A''-DTPA•Tb binding to CL31d / DTPA-R ... 11
Supplementary discussion 2: Modelling of radiation dose caused by [^{18}F]F-DTPA PET to a CAR-T cell using MIRDcell 12
Supplementary Table 4 | Parameters for modelling radiation dose to CAR-T cells using MIRDcell 12
Supplementary Discussion 3: Investigation of increased kidney signals in animals with DTPA-R positive cells.. 14
References 17

Sequences

The gene sequence of the DTPA-R gene along with the translated amino acid sequence.

```
      PacI   10        20        30        40        50        60
    1 CTTAATTAAGCCGCCACCATGCCTCTCGGACTGCTGTGGCTTGGACTGGCTCTGCTGGGA 60
                        Lcn2 signal peptide
                        MetProLeuGlyLeuLeuTrpLeuGlyLeuAlaLeuLeuGly

             70        80        90       100       110       120
   61 GCACTGCATGCTCAGGCCCAGGATAGCACCAGCGATCTGATTCCAGCTCCTCCACTGAGC 120
                        CHX-DTPA•Me binding Anticalin CL31d
      AlaLeuHisAlaGlnAlaGlnAspSerThrSerAspLeuIleProAlaProProLeuSer

            130       140       150       160       170       180
  121 AAGGTGCCCCTGCAGCAGAACTTCCAGGACAACCAATTCCATGGGAAGTGGTATCAAGTG 180
      LysValProLeuGlnGlnAsnPheGlnAspAsnGlnPheHisGlyLysTrpTyrGlnVal

            190       200       210       220       230       240
  181 GGCAGAGCCGGAAATGCCGCTCCTCCTGAAGATCCTGAACTGCTGCTGCTGACAGCCCAG 240
      GlyArgAlaGlyAsnAlaAlaProProGluAspProGluLeuLeuLeuLeuThrAlaGln

            250       260       270       280       290       300
  241 ACCTACGAGCTGAAAGAGGACAAGAGCTACGACGTGACCGCCGTGCGGTTCCGGAAGAAG 300
                                    Asp^85 (no N-glycosylation)
      ThrTyrGluLeuLysGluAspLysSerTyrAspValThrAlaValArgPheArgLysLys

            310       320       330       340       350       360
  301 ATGTGCGAGTACCTGACCATGACCTTCGTGCCTGGATCTCAGCCTGGCGAGTTCACCCTG 360
      MetCysGluTyrLeuThrMetThrPheValProGlySerGlnProGlyGluPheThrLeu

            370       380       390       400       410       420
  361 GGCAACATCAAGAACTACCCCGGCCTGACCAGCTACTTCGTCAGAGTGGTGTCCACCAAC 420
      GlyAsnIleLysAsnTyrProGlyLeuThrSerTyrPheValArgValValSerThrAsn

            430       440       450       460       470       480
  421 TACAACCAGCACGCCATGGTGTTCTTCAAGAAGGTCCAGCAGAACCGCGAGTACTTCAGC 480
      TyrAsnGlnHisAlaMetValPhePheLysLysValGlnGlnAsnArgGluTyrPheSer

            490       500       510       520       530       540
  481 ATCTCTCTGCTCGGCCGGACCAAAGAGCTGACCAGCGAACTGAAAGAGAACTTCATCCGG 540
      IleSerLeuLeuGlyArgThrLysGluLeuThrSerGluLeuLysGluAsnPheIleArg

            550       560       570       580       590       600
  541 TTCTCCAAATCTCTGGGCCTGCCCGAGAATCACATCGTGTTCCCCGTGCCTATCGACCAG 600
      PheSerLysSerLeuGlyLeuProGluAsnHisIleValPheProValProIleAspGln

        ClaI 610       620       630       640       650       660
  601 TGCATCGATGCCAGCGGCAAGCCCATTCCTAATCCTCTGCTGGGCCTCGATTCTACTGGC 660
                     V5-tag
      CysIleAspAlaSerGlyLysProIleProAsnProLeuLeuGlyLeuAspSerThrGly

            670       680       690       700       710       720
  661 GCGCCTGCTTCTCCTGTTCAACCCATGGCTCTGATCGTGCTTGGCGGAGTTGCTGGACTG 720
                  transmembrane domain from CD4
      AlaProAlaSerProValGlnProMetAlaLeuIleValLeuGlyGlyValAlaGlyLeu

            730       740       750       760       770       780
  721 CTGCTGTTTATCGGCCTGGGCATCTTCTTTTGCGTGCGGTGCAGACATAGGCGGAGAGCC 780
      LeuLeuPheIleGlyLeuGlyIlePhePheCysValArgCysArgHisArgArgArgAla

            790 MluI
  781 TCTACCGGTTAAACGCGT 798
      SerThrGlyEnd
```

The gene sequence of the Colchi-R gene is given along with the translated amino acid sequence.

```
      PacI    10         20         30         40         50         60
    1 CTTAATTAAGCCGCCACCATGCCTCTCGGACTGCTGTGGCTTGGACTGGCTCTGCTGGGA 60
                        Lcn2 signal peptide
                        MetProLeuGlyLeuLeuTrpLeuGlyLeuAlaLeuLeuGly

              70         80         90        100        110        120
   61 GCACTGCATGCTCAGGCCCAGGATAGCACCAGCGATCTGATTCCAGCTCCTCCACTGAGC 120
                        CHX-DTPA•Me binding Anticalin CL31d
      AlaLeuHisAlaGlnAlaGlnAspSerThrSerAspLeuIleProAlaProProLeuSer

             130        140        150        160        170        180
  121 AAGGTGCCCCTGCAGCAGAACTTCCAGGACAACCAATTCCATGGGGAGTGGTATGTGGTA 180
      LysValProLeuGlnGlnAsnPheGlnAspAsnGlnPheHisGlyGluTrpTyrValVal

             190        200        210        220        230        240
  181 GGTGTCGCCGGCAATGGTTTCCTCAGAGAAGATAAAGATCCGATTAAAATGGCGGCCACC 240
      GlyValAlaGlyAsnGlyPheLeuArgGluAspLysAspProIleLysMetAlaAlaThr

             250        260        270        280        290        300
  241 ATCTACGAATTAAAAGAAGACAAGTCTTATAACGTGACCTTTATGAAGTTTCCAATGAAG 300
                                  Asn85 allows N-glycosylated
      IleTyrGluLeuLysGluAspLysSerTyrAsnValThrPheMetLysPheProMetLys

             310        320        330        340        350        360
  301 AAATGCGAGTACATGACCGACACGCTGGTTCCTGGAAGTCAGCCCGGCGAGTTTATCCTT 360
      LysCysGluTyrMetThrAspThrLeuValProGlySerGlnProGlyGluPheIleLeu

             370        380        390        400        410        420
  361 GGCAATATCAAGAGTGAACCTGGATACACATCTTGGCTCGTGCGCGTCGTGTCAACAAAC 420
      GlyAsnIleLysSerGluProGlyTyrThrSerTrpLeuValArgValValSerThrAsn

             430        440        450        460        470        480
  421 TATAATCAGCACGCTATGGTGTTCTTTAAGGCTGTTCAACAGAATCGGGAGGACTTTTTC 480
      TyrAsnGlnHisAlaMetValPhePheLysAlaValGlnGlnAsnArgGluAspPhePhe

             490        500        510        520        530        540
  481 ATTACTCTTTACGGAAGGACAATAGAGCTGACCTCCGAGCTGAAGGAGAACTTTATTCGA 540
      IleThrLeuTyrGlyArgThrIleGluLeuThrSerGluLeuLysGluAsnPheIleArg

             550        560        570        580        590        600
  541 TTCTCCAAATCTCTGGGCCTGCCCGAGAATCACATCGTGTTCCCCGTGCCTATCGACCAG 600
      PheSerLysSerLeuGlyLeuProGluAsnHisIleValPheProValProIleAspGln

        ClaI 610        620        630        640        650        660
  601 TGCATCGATGCCAGCGGCAAGCCCATTCCTAATCCTCTGCTGGGCCTCGATTCTACTGGC 660
                     V5-tag
      CysIleAspAlaSerGlyLysProIleProAsnProLeuLeuGlyLeuAspSerThrGly

             670        680        690        700        710        720
  661 GCGCCTGCTTCTCCTGTTCAACCCATGGCTCTGATCGTGCTTGGCGGAGTTGCTGGACTG 720
                  transmembrane domain from CD4
      AlaProAlaSerProValGlnProMetAlaLeuIleValLeuGlyGlyValAlaGlyLeu

             730        740        750        760        770        780
  721 CTGCTGTTTATCGGCCTGGGCATCTTCTTTTGCGTGCGGTGCAGACATAGGCGGAGAGCC 780
      LeuLeuPheIleGlyLeuGlyIlePhePheCysValArgCysArgHisArgArgArgAla

             790 MluI
  781 TCTACCGGTTAAACGCGT 798
      SerThrGlyEnd
```

Supplementary discussion 1: *In silico* analysis of potential immunogenicity of the DTPA-R

To assess the potential immunogenicity of the DTPA-R reporter protein in humans, the sequence was analysed using the web-based IEDB Deimmunization tool (http://tools.iedb.org/deimmunization)[1]. This tool predicts protein cleavage into peptides, loading to HLA variants and their affinities for known T cell receptors. Furthermore, this tool also suggests potential modifications of the input sequence to decrease the immunogenicity of a given peptide, which can help create a de-immunized version of the DTPA-R in the future. The IEDB algorithm, set to the recommended sensitivity threshold, found four different peptides that were considered to potentially cause immunogenicity (**Supplementary Table 1**). The location of these predicted peptides was visualized in an alignment of Lipocalin 2 (UniProt-ID: p80188) and the DTPA-R protein sequence (**Supplementary Fig. 1**). Two of these found peptides (rank 16.0 and 18.465) are located in regions that are 100% identical to the human Lcn2 or CD4 protein sequence. Peptides identically found in endogenous proteins are expected to be non-immunogenic due to tolerance mechanisms of the adaptive immune system for non-foreign epitopes. Furthermore, one peptide (rank 15.0) is in a region that only features a single L=>F exchange compared to the endogenous Lcn2 sequence. The risk for immunogenicity associated with this peptide could be reduced by returning to the Lcn2 sequence, especially as the residue is located at the side of the calyx and points outwards and is not directly involved in ligand binding. Finally, the last predicted peptide (PELLLLTAQTYELKE; rank 17.0) is derived from the loop 1 region of the Anticalin, which is the most mutated region of the Anticalin (comparable with the CDR loops of antibodies). Deimmunization of this region would require protein engineering methods to replace single amino acid residues to decrease potential immunogenicity while preserving or even increasing affinity for CHX-A''-DTPA.

Supplementary Table 1 | Prediction of T cell epitopes from DTPA-R by the IEDB.org immunogenicity tool

Protein	Start Position	End Position	Median Percentile Rank	Peptide
DTPA-R	101	115	15.0	PGLTSYFVRVVSTNY
	151	165	16.0	NFIRFSKSLGLPENH
	46	60	17.0	PELLLLTAQTYELKE
	211	225	18.465	VAGLLLFIGLGIFFC

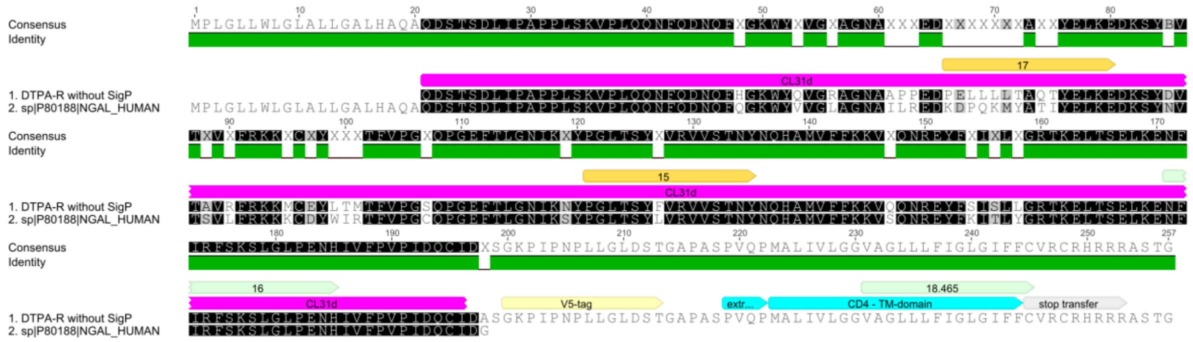

Supplementary Fig. 1 | Analysis of potential peptides derived from the immunoproteasome and HLA•peptide complexes. Four potentially immunogenic peptides (labelled with their score: 17, 15, 16, and 18.465) were identified by the IEDB Deimmunization algorithm. To illustrate the sequence context of these potentially immunogenic peptides, they were annotated in an alignment of the human Lcn2/NGAL and the DTPA-R protein sequences.

Supplementary Table 2 | Antibodies & staining reagents

Antibody / reactivity	Supplier	Catalog number	Conjugation	Clone name	Lot number	Application
anti-V5-tag (murine)	Bio-Rad	MCA1360	none or AF488	SV5-Pk1	150547,148239	IF, FACS, MACS, IHC(P), WB
anti-β-actin	Bio-Rad	discontinued	Dylight CW680	AbD12141	0114	WB
anti-mouse IgG	LI-COR	926-32210	IRDye 800CW	n.a.	C91210-09	WB
anti-mouse IgG $[F(ab')_2]$	Invitrogen	A21204	AF488	n.a.	2155587	IF
anti-human EGFR(t)	BioLegend	352904	PE	AY13	B336514	FACS
anti-V5-tag	Life Technologies	12-6796-42	PE	TCM5	2301156	FACS
anti-human CD3	Life Technologies	17-0038-42	APC	UCHT1	2376138	FACS
anti-human CD8	eBioscience	47-0086-42	APC-efluor780	OKT8	2611767	FACS
anti-human CD45	Beckman Coulter	B36294	krome orange (KO)	J33	200097	FACS
Streptavidin (binds *Strep*-tag within CAR)	Life Technologies	48-4317-82	efluor450	n.a.	2527387	FACS
Streptavidin	Biolegend	405201	FITC	n.a.	B309228	FACS
anti-CD8	eBioscience	12-0086-42	PE	OKT8	2504398	FACS
anti-CD8	BioLegend	301049	APC	RPA-T8	B368721	FACS
anti-CD8	eBioscience	47-0086-42	APC-efluor780	OKT8	2611767	FACS
anti-CD8	Life Technologies	MHCD0830	pacific orange ~ KO	3B5	2375611	FACS
anti-CD8	eBioscience	48-0086-42	efluor450	OKT8	2410932	FACS
anti-CD69	BioLegend	310910	APC	FN50	B337763	FACS
anti-CD3	BioLegend	300319	AF488	HIT3a	B278329	FACS
anti-CD4	BioLegend	317419	AF488	OKT4	B292040	FACS
anti-CXCR3 / CD183	BioLegend	353709	AF488	G025H7	B264198	FACS
anti-human CD19	Cell Signaling Technology	90176S	none	D4V4B	1	IHC(P)

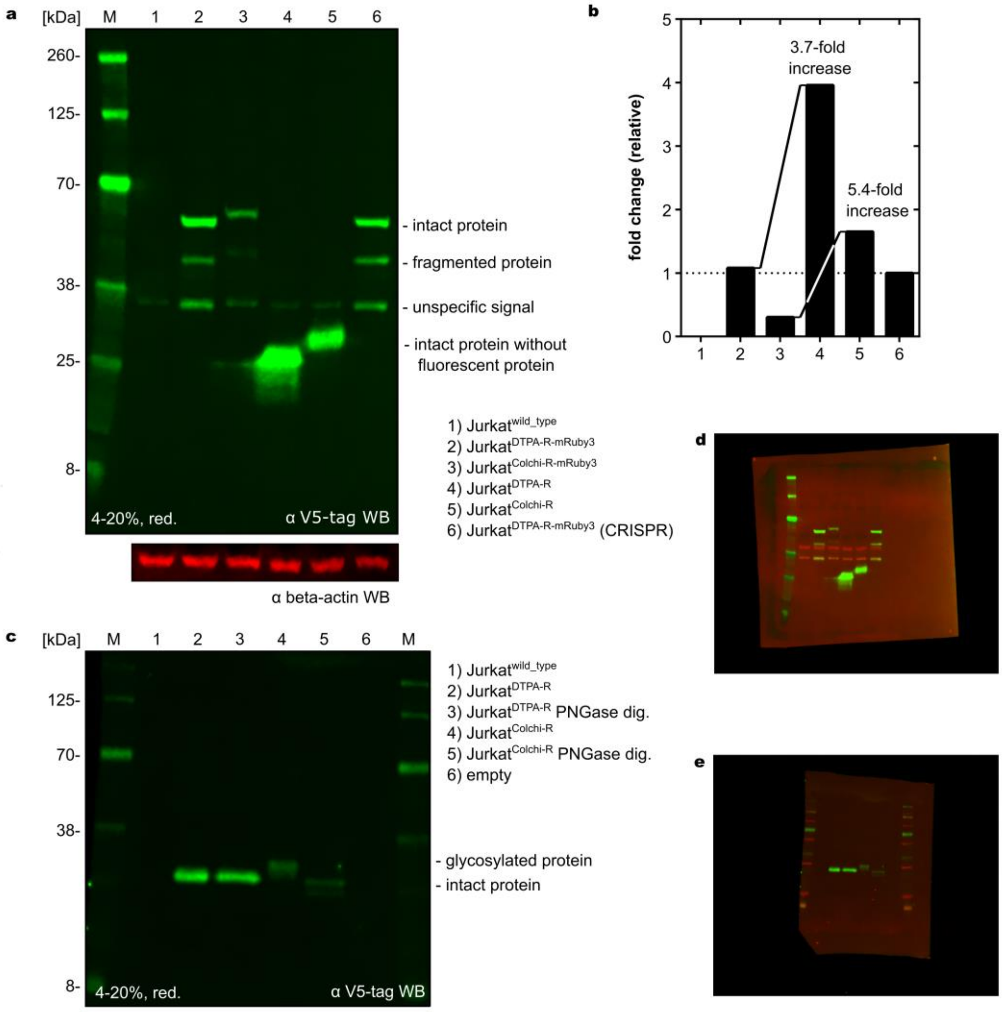

Supplementary Fig. 2 | Reporter protein cleavage studies using anti-V5 Western blot analysis. a, Jurkat cell lines transduced with different versions of the reporter genes DTPA-R and Colchi-R were cultured and lysed using RIPA-buffer. After electrophoretic separation by SDS-PAGE, a Western blot was conducted against the V5-tag and beta-actin as a loading control. While reporter proteins featuring a mRuby3 fluorescent protein showed an additional band indicating a potential proteolytic fragmentation, reporter proteins with no fluorescent protein were higher expressed and showed no unexpected bands. In all lanes, an unspecific band, presumably a protein from the Jurkat cell, was detected. DTPA-R bands migrated slightly lower compared to Colchi-R, which can be explained by the loss of the N-glycosylation site of Lcn2 in the coding sequence of DTPA-R but not in Colchi-R. **b**, Quantification of fluorescence signals of individual bands allowed the quantitative comparison of relative expression levels. Expression levels were increased by omitting the intracellular fluorescent protein and using DTPA-R over Colchi-R. **c**, Western blot analysis of DTPA-R and Colchi-R reporter protein before and after enzymatic removal of N-linked oligosaccharides by PNGase F. Larger molecular size before digestion and clear down-shift after PNGase F treatment indicate N-glycosylation, presumably at Asn^{85} (natural glycosylation site within Lcn2)[2] in Colchi-R but not DTPA-R (where this amino acid was mutated to Asp^{85}). **d,e**, Complete Western blots of (**a**) and (**c**) respectively.

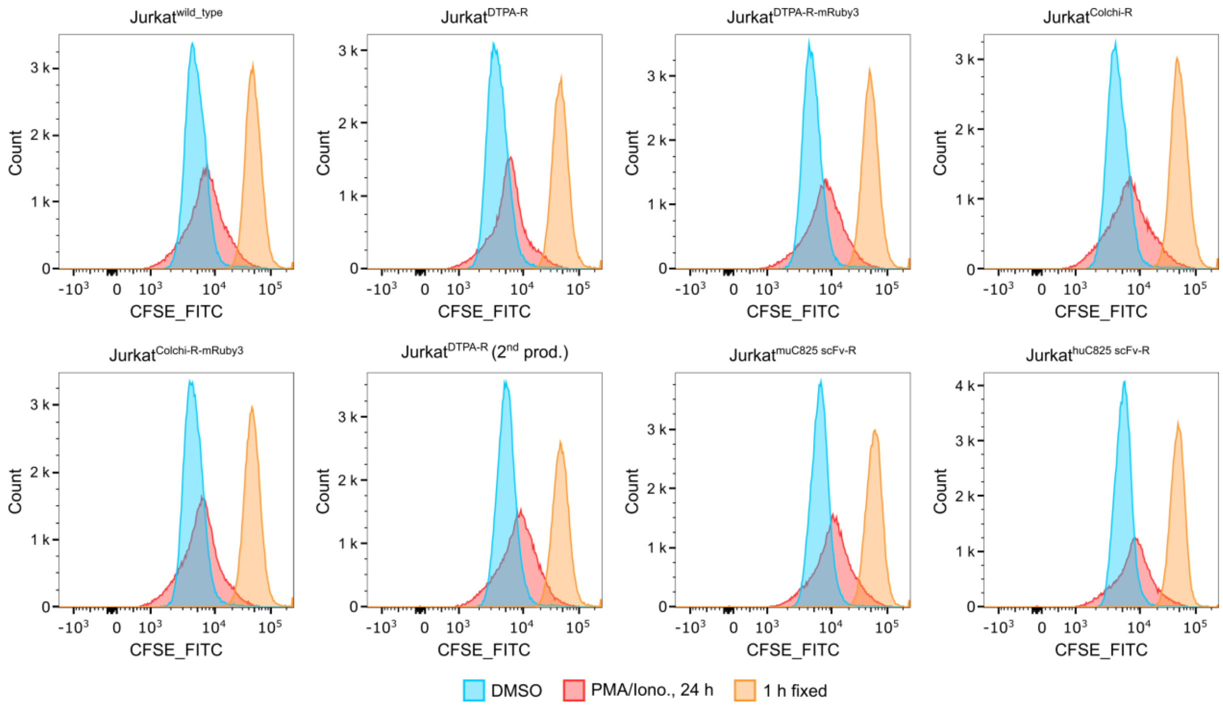

Supplementary Fig. 3 | Raw data for CFSE proliferation assay. Jurkat cell lines transduced with different versions of the reporter genes DTPA-R and Colchi-R, or the C825 scFv-receptor, were stained with CFSE and either fixed after 1 h (orange) or cultured for 3 days in cell culture medium containing either PMA and Ionomycin (PMA/Iono.) for activation (red) or control medium with equivalent DMSO (blue). Cells were analysed by flow cytometry and the doubling time for the individual cells was calculated.

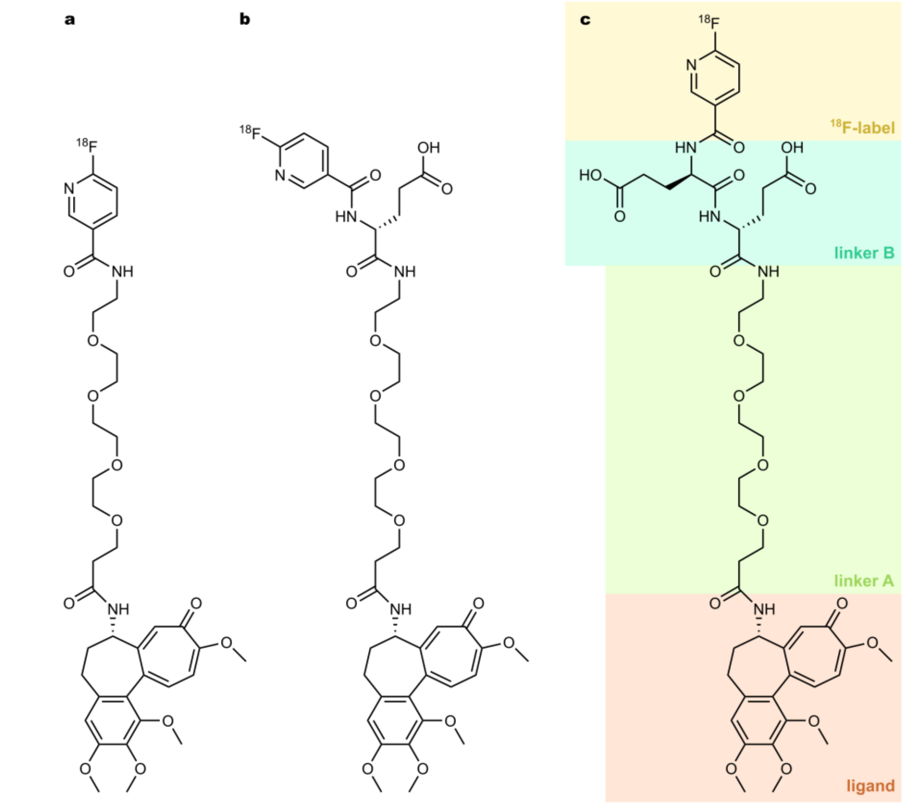

[^{18}F]F-Nic-PEG$_4$-colchicine
exact mass: 726.31 Da
charge (pH 7.4) = 0
miLogP (predict.) = 0.79
LogD$_{7.4}$ (experiment) = 0.89
=> 11.4% in PBS phase

[^{18}F]F-Nic-Glu-PEG$_4$-colchicine
exact mass: 855.36 Da
charge (pH 7.4) = -1
miLogP (predict.) = -0.33
LogD$_{7.4}$ (experiment) = -1.92
=> 98.8% in PBS phase

[^{18}F]F-Nic-Glu$_2$-PEG$_4$-colchicine
exact mass: 984.40 Da
charge (pH 7.4) = -2
miLogP (predict.) = -1.45
LogD$_{7.4}$ (experiment) = -2.97
=> 99.9% in PBS phase

Supplementary Fig. 4 | Hydrophilicity engineering of [^{18}F]F-Nic-PEG$_4$-colchicine. a–c, Radioligands for indirect targeting of colchicine-binding reporter protein Colchi-R. The design includes a nicotinic acid with an ^{18}F-label, a PEG$_4$ linker, and the colchicine molecule, which functions as a ligand moiety bound by the Anticalin. Initial radioligand (**a**) without additional hydrophilic amino acid, (**b**) with one or (**c**) with two D-Glu residues. Besides the chemical structure, the charge at physiological pH, a predicted partitioning coefficient (miLogP; www.molinspiration.com/cgi-bin/properties), and the experimentally determined LogD$_{7.4}$ are given.

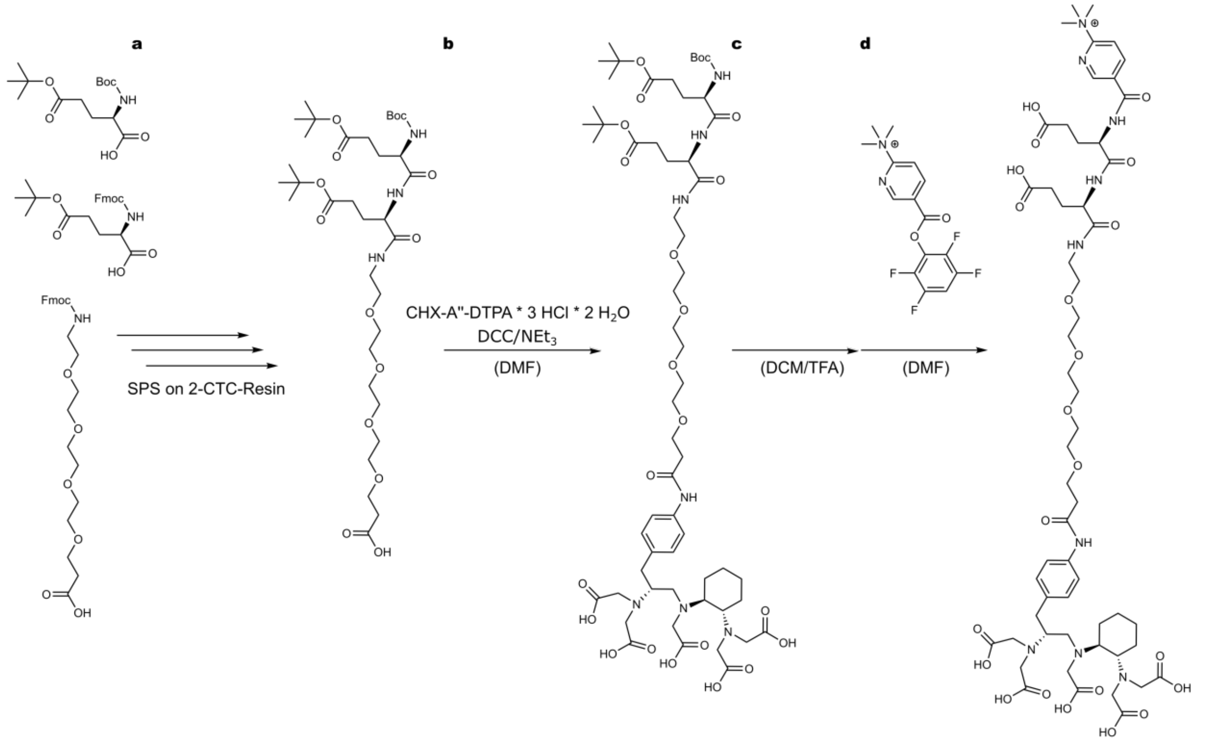

Supplementary Fig. 5 | Preparation of TMA-Py-Glu₂-PEG₄-CHX-A''-DTPA precursor. a, Solid Phase Synthesis (SPS) on 2-CTC resin following standard protocol. **b**, Coupling of the linker to CHX-A''-DTPA (commercially available from Macrocylics, Plano, TX) with Dicyclohexylcarbodiimid (DCC) in DMF, r.t. for 18 h. **c**, Deprotection in TFA/DCM (1:1) at r.t. for 5 h. **d**, The activated Nicotinic acid (Nic) ester was prepared according to the literature protocol[3], coupling was performed in DMF at r.t. for 18 h.

Supplementary Table 3 | Affinity measurements of CHX-A''-DTPA•Tb binding to CL31d / DTPA-R

Method	Compound	IC_{50} / K_D [M]	k_{on} [$M^{-1}\cdot s^{-1}$]	k_{off} [$1\cdot s^{-1}$]
Realtime Interaction Cytometry (RT-IC)	AlexaFluor647-PEG_4-[thiourea]-CHX-A''-DTPA•^{nat}Tb<br>cf. Extended Data Fig. 5d–h	3.80×10^{-10}	1.60×10^{6}	6.08×10^{-4}
		4.64×10^{-10}	1.32×10^{6}	6.12×10^{-4}
		4.18×10^{-10}	1.42×10^{6}	5.94×10^{-4}
		Average: $4.21\times10^{-10}\pm3.42\times10^{-11}$	Average: $1.45\times10^{6}\pm1.16\times10^{5}$	Average: $6.05\times10^{-4}\pm7.72\times10^{-6}$
	AlexaFluor647-PEG_4-[peptide]-CHX-A''-DTPA•^{nat}Tb<br>cf. Extended Data Fig. 5i–k	3.00×10^{-10}	1.32×10^{6}	3.97×10^{-4}
		7.20×10^{-10}	7.94×10^{5}	5.74×10^{-4}
		4.62×10^{-10}	1.38×10^{6}	6.36×10^{-4}
		6.03×10^{-10}	7.19×10^{5}	4.33×10^{-4}
		4.48×10^{-10}	9.89×10^{5}	4.43×10^{-4}
		Average: $5.07\times10^{-10}\pm1.43\times10^{-10}$	Average: $1.04\times10^{6}\pm2.65\times10^{5}$	Average: $4.97\times10^{-4}\pm9.19\times10^{-5}$
Surface plasmon resonance (SPR) spectroscopy	eGFP-[thiourea]-CHX-A''-DTPA•^{nat}Tb<br>cf. Extended Data Fig. 5l	6.14×10^{-10}	4.86×10^{5}	2.99×10^{-4}
Competitive binding assay (IC_{50})	NH_2-CHX-A''-DTPA•^{nat}Tb<br>cf. Extended Data Fig. 5m–o, 5s-t	3.32×10^{-10}	Not applicable	
		2.13×10^{-10}		
		1.96×10^{-10}		
		Average: $2.47\times10^{-10}\pm6.05\times10^{-11}$		
	^{19}F-Glu_2-PEG_4-CHX-A''-DTPA•^{nat}Tb (≙ [^{18}F]F-DTPA)<br>cf. Extended Data Fig. 5p–r, 5u–v	5.05×10^{-10}		
		1.65×10^{-10}		
		2.26×10^{-10}		
		Average: $2.99\times10^{-10}\pm1.48\times10^{-10}$		

Supplementary discussion 2: Modelling of radiation dose caused by [^{18}F]F-DTPA PET to a CAR-T cell using MIRDcell

During a DTPA-R PET scan, cells expressing the DTPA-R reporter gene are exposed to an elevated radiation dose by binding radioactive [^{18}F]F-DTPA. The radiation dose to the CAR-T cells is almost exclusively due to the emitted positrons which have a range of about 0.6 mm[4, 5]. The contribution is of the 511 keV annihilation photons is comparably negligible[4, 5]. Zanzonico et al.[5] have extensively studied the effects of radiolabelling on the function of lymphocytes. They found no significant effects on lymphocyte function for radiation doses of up to 8.3 Gy from ^{131}I-FIAU. For external beam radiotherapy, toxic effects have been observed at much lower doses (see review by Harald Paganetti)[6]. However, these studies have used much higher dose rates than the ones produced by radionuclides used in nuclear medicine. We used MIRDcell V3.13 software[7, 8] to determine the maximum radiation dose to CAR T cells by a PET scan. MIRDcell calculates dosimetry for individual cells or clusters of cells considering self and cross fire doses (i.e, radiation dose received from positrons emitted by neighbouring cells). The ^{18}F full beta+ energy spectrum was used with the cell and nucleus radius of a Jurkat cell (8 µm and 6 µm nucleus)[9], assuming 90% surface-bound [^{18}F]F-DTPA, as measured in **Fig. 2d**. Based on the correlation of measured PET signals and CAR-T cell numbers *in vivo* (**Fig. 6b**), and phantom studies with Jurkat$^{\text{DTPA-R}}$ cells (**Fig. 6e**), we estimated the maximal activity per cell to be 0.04 Bq assuming clusters of 12,500 cells. We also conservatively assumed that there is no biological clearance of the ^{18}F from the CAR-T cells. The resulting self dose of a cell was 0.037 Gy, a value that is 224-fold below the threshold reported by Zanzonico et al.[5].

However, the T cells also appear in clusters (e.g., at the tumour sites), and are therefore exposed to a cross dose from neighbouring cells, which can result in higher radiation doses per cell. The dose due to cross fire is highest in the centre of the cluster and decreases for cells at the periphery as shown in **Supplementary Fig. 6a**. For further analysis we therefore used the median dose for the cells in a cluster (**Supplementary Fig. 6b**) to describe radiation dose to CAR T cells. First, we studied the impact of the percentages of labelled cells in a cluster and found that median dose increases linearly with the percentage of labelled cells (**Supplementary Fig. 6c**). Furthermore, the impact of the size of a cell cluster was evaluated. This calculation showed that median radiation doses ranged from of 0.086 Gy (0.1 mm) to up to 0.714 Gy (1 mm) (**Supplementary Fig. 6d**). All these calculations were made with the conservative assumption that the uptake per cell is 0.04 Bq and that there is no biological clearance. Comparing the self-absorbed dose values for different radionuclides showed comparable low self doses for ^{18}F-labeled cells (7.46×10^{-5} Gy/B/s), while other nuclides lead to higher self doses (1.74×10^{-4} Gy/Bq/s for ^{89}Zr or 2.54×10^{-4} Gy/Bq/s for ^{111}In) (**Supplementary Fig. 6e**). Notably, the median dose for a cluster of cells differs to a greater extent with higher doses for nuclides like ^{111}In and ^{89}Zr and lower doses for e.g. ^{68}Ga and ^{18}F (**Supplementary Fig. 6f**). Also, cross doses of ^{18}F were low when compared to other diagnostic isotopes used in nuclear medicine. In summary, the radiation dose for [^{18}F]F-DTPA is well below the radiation toxicity level for lymphocytes at 830 cGy (8.3 Gy)[5] and lower than most of the commonly used diagnostic isotopes. These comparisons assume full physical decay of the respective isotope. Other parameters used for the calculations are summarized in Supplementary Table 4.

Supplementary Table 4 | Parameters for modelling radiation dose to CAR-T cells using MIRDcell

	Parameter	Value
General	Cell radius	8 µm
	Nucleus radius	6 µm
	% of activity on surface/cytosol	90%/10%
	Distance between cells	16 µm
3D cluster	Radius	50-500 µm
	Labelling	Uniform distribution
	Mean activity per cell	0.04 Bq

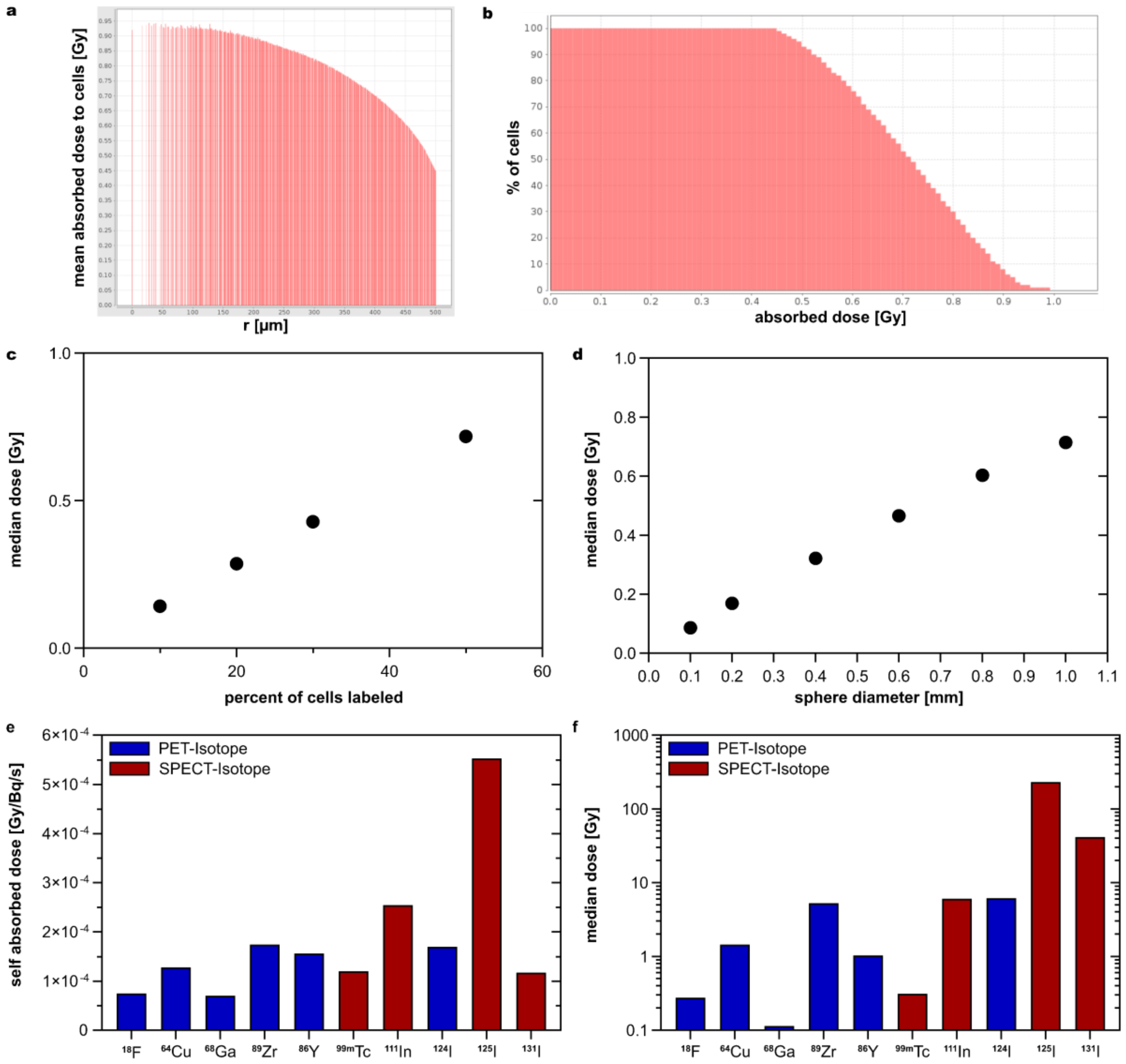

Supplementary Fig. 6 | Modelling of the radiation dose caused by [^{18}F]F-DTPA PET scan to a CAR-T cell using MIRDcell software. Dose estimations for cells with 8 µm radius and 6 µm nucleus in a 1 mm sphere and 50% of all cells labelled (**a**,**b**). **a**, Mean absorbed dose for cells depends on the distance of the cell from the centre. **b**, Percentage of cells exposed to different radiation doses. **c**, Correlation of percentage of cells labelled in a 1 mm sphere with the median absorbed dose by ^{18}F. **d**, Correlation of the sphere diameter (50% of the cells ^{18}F labelled) and the median absorbed dose. **e**,**f**, Comparison of self-absorbed dose (**e**) and median dose (**f**) caused by different radionuclides in a sphere with 200 µm diameter and 100% labelled cells.

Supplementary Discussion 3: Investigation of increased kidney signals in animals with DTPA-R positive cells

It was observed that mice with an increased number of cells expressing DTPA-R in the periphery showed increased kidney signals in static [^{18}F]F-DTPA PET scans, which was the case for DTPA-R expression in murine cells upon AAV9 transduction (**Fig. 7e and Supplementary Fig. 7a**) and infiltration of CAR-T$^{\text{DTPA-R}}$ cells (**Extended Data Fig. 6 and Supplementary Fig. 7b**). The gradually increased kidney signal correlated well with the amount of AAV9 dose (R^2=0.87) (**Supplementary Fig. 7a**) and peripherally expressed DTPA-R on CAR-T cells (R^2=0.66) while control animals (CAR-T$^{\text{EGFR-T}}$) showed low kidney and whole body signal (**Supplementary Fig. 7b**). The retained activity in the muscle of the CAR-T mice did not correlate with the kidney values (R^2=0.02), indicating that there is no impairment in kidney function (**Supplementary Fig. 7c,** #746). To analyse direct elimination kinetics following i.v. injection of [^{18}F]F-DTPA, a dynamic PET scan of a wild type mouse (female C57BL/6, three months old) that does not possess any cells expressing DTPA-R was analysed (**Supplementary Fig. 7d**). A time-activity curve was generated based on a threshold segmentation with an isocontour at the 90% maximal kidney signal (**Supplementary Fig. 7e**). Direct comparison of the last time frames (t=75**–**90 min) of the wild type mouse (#792) (**Supplementary Fig. 7d**) and a mouse with a PC3$^{\text{DTPA-R}}$ xenograft tumour (#655) (**Supplementary Fig. 7f**) showed a marked difference in the kidney signal confirming previous findings. However, visual and quantitative analysis of the PET images revealed that mice harbouring DTPA-R expressing cells retained substantial amounts of radioactivity in the renal cortex, whereas the radioactivity was quickly cleared to the pelvis in control animals (**Supplementary Fig. 7d,g-j**).

It is well established that Anticalin proteins labelled with radiometals are rapidly accumulated and retained in the renal cortex of mice[10, 11]. Following i.v. injection typical activity concentrations are in the order of 100 %ID/g in the kidney with only slow clearance over time. We therefore hypothesized that the correlation between renal activity and the number of DTPA-R expressing cells is due to binding of [^{18}F]F-DTPA to shedded DTPA-R. The shedded DTPA-R/[^{18}F]F-DTPA would be rapidly cleared from the blood pool by the kidneys and would remain in the kidney at the time of imaging (90 min p.i.).

We therefore investigated *in vitro* if cells transduced with DTPA-R shed the ectodomain protein into the cell culture media. By Western blot analysis we detected DTPA-R fragments in the culture media that still contained the V5-tag (**Supplementary Fig. 7k**) and retained their ability to bind [^{18}F]F-DTPA (**Supplementary Fig. 7l**). These data indicate that shedded DTPA-R/[^{18}F]F-DTPA complexes may explain the increased radioactivity concentration in the cortex of mice harbouring DTPA-R expressing cells. However, the maximal kidney uptake of [^{18}F]F-DTPA in mice with CAR-T$^{\text{DTPA-R}}$ cells is 16.3 %ID/g and therefore below various other clinically used radioligands (e.g. 84-142 %ID/g for ^{18}F-PSMA-1007[12]) indicating that this may not be a clinically relevant limitation.

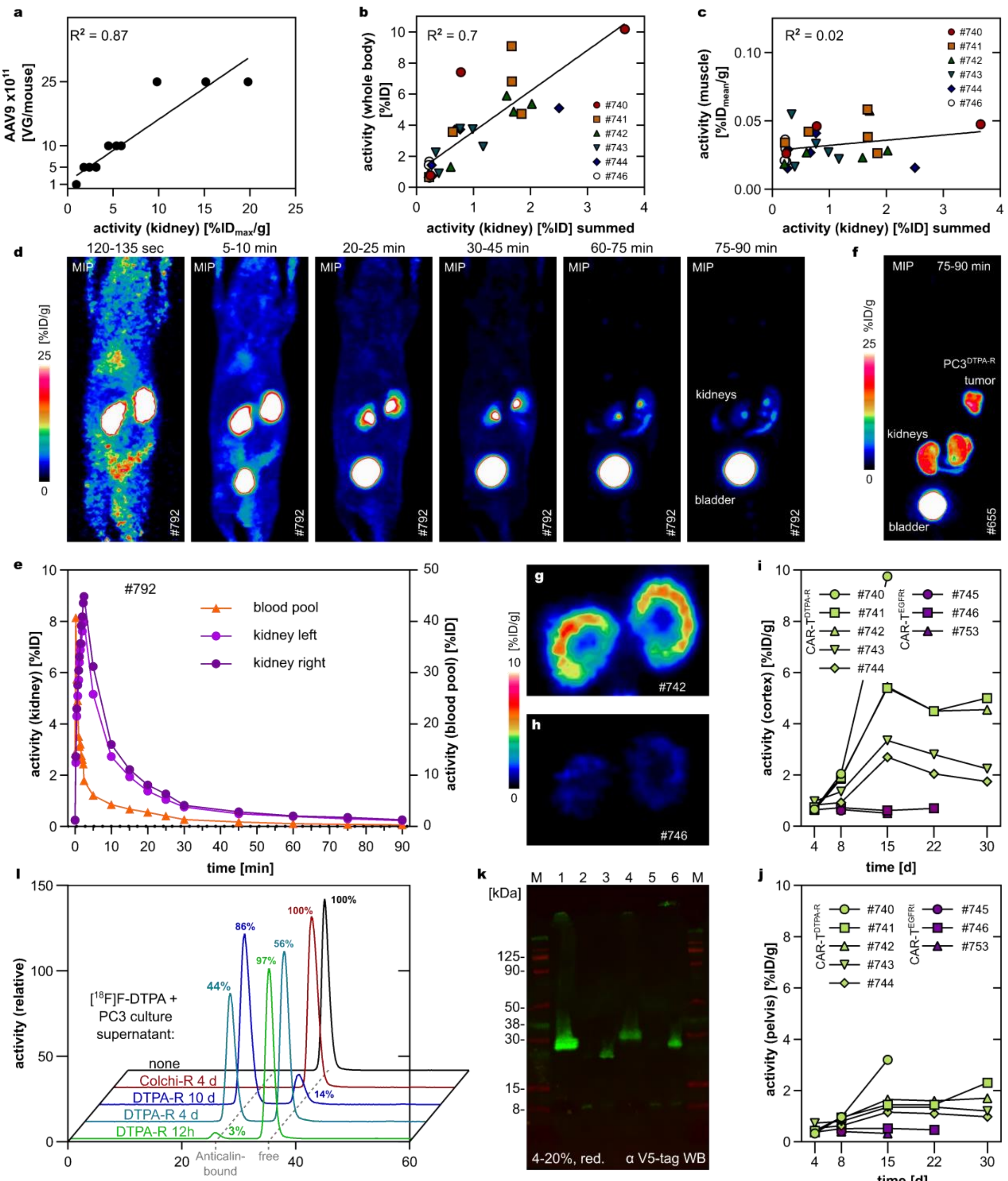

Supplementary Fig. 7 | DTPA-R expressing cells in the periphery lead to increased kidney signals. a, In the AAV9 cohort, the increase in kidney signal correlated well with increasing viral doses. **b**, In the CAR-T cell imaging cohort (cf. **Fig. 5**) increased kidney signals correlated with the increase in specific [^{18}F]F-DTPA uptake in the periphery (not including unspecific signals in the abdomen). **c**, In contrast, the signal in muscle tissue (probed with a sphere in the triceps and biceps) in CAR-T mice was not increasing with increasing kidney signal, indicating the absence of kidney failure. **d**, Dynamic PET scan of a female wild type C57BL/6 mouse injected i.v. with [^{18}F]F-DTPA over 90 min, and **e**, time-activity curve of kidneys and blood pool (assuming 6% blood volume and a 25g mouse). **f**, Last time frame (t=75–90 min) of the dynamic PET scan of a CD1-nude mice with subcutaneous PC3$^{DTPA\text{-}R}$ (right shoulder) and PC3$^{Colchi\text{-}R}$ (left shoulder) tumour injected i.v. with [^{18}F]F-DTPA as depicted in Fig. 3a. **g,h**, Coronal kidney slices of CAR-T$^{DTPA\text{-}R}$ mouse (**g**) and CAR-T^{EGFRt} mouse (**h**) showing retention of radioligand specifically in the kidney cortex. **i,j**, quantification of PET-

signal in the renal cortex (**i**) and the pelvis (**j**) of CAR-T mice during treatment. **k**, Western blot analysis of cell culture supernatant stained with the anti-V5-tag antibody (lanes: 1–PC3$^{DTPA\text{-}R}$ lysate; 2–PC3$^{DTPA\text{-}R}$ supernatant 12 h; 3–PC3$^{DTPA\text{-}R}$ supernatant 4 d; 4–PC3$^{Colchi\text{-}R}$ lysate; 5–PC3$^{Colchi\text{-}R}$ supernatant 12 h; 6–PC3$^{Colchi\text{-}R}$ supernatant 4 d). **l**, Size exclusion chromatography of cell culture supernatant from PC3$^{DTPA\text{-}R}$ and PC3$^{Colchi\text{-}R}$ cells incubated with [^{18}F]F-DTPA.

References

1. Dhanda, S.K. et al. Development of a strategy and computational application to select candidate protein analogues with reduced HLA binding and immunogenicity. *Immunology* **153**, 118-132 (2018).
2. Kjeldsen, L., Johnsen, A.H., Sengelov, H. & Borregaard, N. Isolation and primary structure of NGAL, a novel protein associated with human neutrophil gelatinase. *J Biol Chem* **268**, 10425-10432 (1993).
3. Feiner, I.V.J. et al. Pre-targeting with ultra-small nanoparticles: boron carbon dots as drug candidates for boron neutron capture therapy. *J Mater Chem B* **9**, 410-420 (2021).
4. Conti, M. & Eriksson, L. Physics of pure and non-pure positron emitters for PET: a review and a discussion. *EJNMMI Phys* **3**, 8 (2016).
5. Zanzonico, P. et al. [^{131}I]FIAU labeling of genetically transduced, tumor-reactive lymphocytes: cell-level dosimetry and dose-dependent toxicity. *Eur J Nucl Med Mol Imaging* **33**, 988-997 (2006).
6. Paganetti, H. A review on lymphocyte radiosensitivity and its impact on radiotherapy. *Front Oncol* **13**, 1201500 (2023).
7. Vaziri, B. et al. MIRD pamphlet No. 25: MIRDcell V2.0 software tool for dosimetric analysis of biologic response of multicellular populations. *J Nucl Med* **55**, 1557-1564 (2014).
8. Katugampola, S., Wang, J., Rosen, A., Howell, R.W. & committee, S.M. MIRD Pamphlet No. 27: MIRDcell V3, a Revised Software Tool for Multicellular Dosimetry and Bioeffect Modeling. *J Nucl Med* **63**, 1441-1449 (2022).
9. Morath, V. et al. Semi-automatic determination of cell surface areas used in systems biology. *Frontiers in bioscience* **5**, 533-545 (2013).
10. Deuschle, F.C. et al. Development of a high affinity Anticalin((R)) directed against human CD98hc for theranostic applications. *Theranostics* **10**, 2172-2187 (2020).
11. Morath, V. et al. Molecular Design of ^{68}Ga- and ^{89}Zr-Labeled Anticalin Radioligands for PET-Imaging of PSMA-Positive Tumors. *Mol Pharm* **20**, 2490-2501 (2023).
12. Cardinale, J. et al. Preclinical Evaluation of ^{18}F-PSMA-1007, a New Prostate-Specific Membrane Antigen Ligand for Prostate Cancer Imaging. *J Nucl Med* **58**, 425-431 (2017).
