# Supplementary material for: PET-based tracking of CAR T cells and viral gene transfer using a cell surface reporter that binds to lanthanide complexes
Source: Nat Biomed Eng. 2025 Jun 13;9(11):1886–906. doi: 10.1038/s41551-025-01415-7 (PMC12623248; doi:10.1038/s41551-025-01415-7)
Supplement: Supplementary file 2 — Reporting Summary [file 41551_2025_1415_MOESM2_ESM.pdf]

nature portfolio

Corresponding author(s): Wolfgang A. Weber

Last updated by author(s): 04 June 2025

Reporting Summary

Nature Portfolio wishes to improve the reproducibility of the work that we publish. This form provides structure for consistency and transparency in reporting. For further information on Nature Portfolio policies, see our Editorial Policies and the Editorial Policy Checklist.

Statistics

For all statistical analyses, confirm that the following items are present in the figure legend, table legend, main text, or Methods section.

n/a	Confirmed	
☐	☒	The exact sample size (*n*) for each experimental group/condition, given as a discrete number and unit of measurement
☐	☒	A statement on whether measurements were taken from distinct samples or whether the same sample was measured repeatedly
☐	☒	The statistical test(s) used AND whether they are one- or two-sided<br>*Only common tests should be described solely by name; describe more complex techniques in the Methods section.*
☒	☐	A description of all covariates tested
☐	☒	A description of any assumptions or corrections, such as tests of normality and adjustment for multiple comparisons
☐	☒	A full description of the statistical parameters including central tendency (e.g. means) or other basic estimates (e.g. regression coefficient) AND variation (e.g. standard deviation) or associated estimates of uncertainty (e.g. confidence intervals)
☐	☒	For null hypothesis testing, the test statistic (e.g. *F*, *t*, *r*) with confidence intervals, effect sizes, degrees of freedom and *P* value noted<br>*Give P values as exact values whenever suitable.*
☒	☐	For Bayesian analysis, information on the choice of priors and Markov chain Monte Carlo settings
☒	☐	For hierarchical and complex designs, identification of the appropriate level for tests and full reporting of outcomes
☒	☐	Estimates of effect sizes (e.g. Cohen's *d*, Pearson's *r*), indicating how they were calculated

Our web collection on statistics for biologists contains articles on many of the points above.

Software and code

Policy information about availability of computer code

Data collection	PET data were collected using a nanoScan PET/MR system with 3T field strength and two PET rings (Mediso Medical Imaging Solutions, Budapest, Hungary). The scanner was operated using the Nucline NanoScan software (ver. 3.04.025.0000).
Data analysis	Reconstructed PET data were analyzed using the Inveon Research Workplace software (ver. 4.2; Siemens Medical Solutions, Knoxville, TN) by 3D isocontour set at 50% of the maximum intensity voxel, spheres with a diameter of 10 to 40 pixels or by 3D volume of interest (VOI) calculating %ID/g or activity.<br>Various data were analyzed and plotted using Prism software (ver. 9.3.1; GraphPad, San Diego, CA).<br>Histology data were visualized using Aperio ImageScope software (ver. 12.4; Leica Biosystems). The positive cell fraction of IHC(P) results was analyzed using the QuPath software (ver. 0.3.2).<br>Flow cytometry results were analyzed using the FlowJo software (ver. 10.8.1; Becton Dickinson).<br>xCELLigence data were analyzed using the RTCA software pro (ver. 2.0.0.1301; ACEA Bioscience).<br>Chemical structures were visualized using ChemDraw (ver. 21.0.0; PerkinElmer)<br>Radiation dose was modelled using MIRDcell software (ver. 3.13; MIRDsoft)

For manuscripts utilizing custom algorithms or software that are central to the research but not yet described in published literature, software must be made available to editors and reviewers. We strongly encourage code deposition in a community repository (e.g. GitHub). See the Nature Portfolio guidelines for submitting code & software for further information.

Data

Policy information about availability of data

All manuscripts must include a data availability statement. This statement should provide the following information, where applicable:

- Accession codes, unique identifiers, or web links for publicly available datasets
- A description of any restrictions on data availability
- For clinical datasets or third party data, please ensure that the statement adheres to our policy

The authors declare that the main data supporting the findings of this study are available within the publication and its Supplementary Information files. The corresponding author will make raw data and step-by-step protocols available upon request. However, the source data for all figures is available.

Research involving human participants, their data, or biological material

Policy information about studies with human participants or human data. See also policy information about sex, gender (identity/presentation), and sexual orientation and race, ethnicity and racism.

Reporting on sex and gender	not applicable
Reporting on race, ethnicity, or other socially relevant groupings	not applicable
Population characteristics	not applicable
Recruitment	not applicable
Ethics oversight	not applicable

Note that full information on the approval of the study protocol must also be provided in the manuscript.

Field-specific reporting

Please select the one below that is the best fit for your research. If you are not sure, read the appropriate sections before making your selection.

☒ Life sciences ☐ Behavioural & social sciences ☐ Ecological, evolutionary & environmental sciences

For a reference copy of the document with all sections, see nature.com/documents/nr-reporting-summary-flat.pdf

Life sciences study design

All studies must disclose on these points even when the disclosure is negative.

Sample size	The sample size used depends on the particular experiment. No sample-size calculations were performed. For in vivo PET/MR imaging studies, the sample size was limited by the number of animals that could be measured in the scanner in one day. For the AAV9 studies, n=3 mice per cohort were used, which allowed the measurement of two cohorts per day. For the CAR-T study presented, n=6 mice were used for the longitudinal cohort and n=6 animals for the endpoint cohort. Both cohorts were shifted by one day to overcome the limiting PET/MR scan time while using the same batch of tumor and CAR-T cells for this study to ensure comparability. For the CAR-T cell treatment study comparing CAR-T cells expressing DTPA-R with those expressing EGFRt, the sample size (n=5 vs. 5 + 3 control) was chosen according to institutional protocols and was limited by the maximum number of animals allowed in the initial orientation studies according to the German animal welfare regulations.<br>Sample size for in vitro assays was chosen based on literature and on established institutional protocols. In vitro assays were usually performed in triplicates and reproduced in biological and/or technical replications, as indicated in the figure legends. For flow cytometry, typically, a number of 100,000 events was analyzed per stained sample.
Data exclusions	PET signals caused by external contamination of the animal were not included in the data evaluation (mouse #741). Apart from this, no animals were excluded. Data from in vitro assays were not excluded except for the border wells in the xCelligence killing assay (lane A and H and column 1 and 12).
Replication	In vitro experiments were mainly done in duplicates, triplicates or quadruplicates (technical replication) to control for experimental variation, and experiments were repeated to show the reproducibility of the findings (biological replication). Figure panels either show results from different experiments (e.g., Fig. 1f) or from a representative experiment (e.g., Fig. 1h or Fig. 2a).<br>Animal studies were conducted with a predefined number of animals as outlined in the approved animal experimentation license. According to the German animal protection act, double or repeated animal studies are not permitted (§ 8 Abs. 3 TierSchG).
Randomization	Animals were randomly assigned to treatment and control groups.
Blinding	For the preclinical experiments, investigators were not blinded to the study groups. Due to local genetic engineering regulations, it is

Blinding	necessary to unmistakably label GMOs and animals carrying them. Outcome measures were primarily imaging data, which was recorded by the scanner in an unbiased fashion, survival (only for CAR-T), and ex vivo analysis (ddPCR, IHC, biodistribution analysis). The outcome parameter survival was unsuspicious to be unbiased as animals reached the predefined humane endpoint by body weight loss, which is an objective, unbiased parameter. The ex vivo analysis (ddPCR, IHC) was evaluated in a blinded manner (collaborators from different institutions). Due to the kind of study and its outcome parameters, it is highly unlikely that a non-blinded researcher consciously or unconsciously influenced the study's outcome.<br><br>Quantitative data analysis and validation controls were used, minimizing the risk of introducing bias through the absence of blinding.

Behavioural & social sciences study design

All studies must disclose on these points even when the disclosure is negative.

Study description	*Briefly describe the study type including whether data are quantitative, qualitative, or mixed-methods (e.g. qualitative cross-sectional, quantitative experimental, mixed-methods case study).*
Research sample	*State the research sample (e.g. Harvard university undergraduates, villagers in rural India) and provide relevant demographic information (e.g. age, sex) and indicate whether the sample is representative. Provide a rationale for the study sample chosen. For studies involving existing datasets, please describe the dataset and source.*
Sampling strategy	*Describe the sampling procedure (e.g. random, snowball, stratified, convenience). Describe the statistical methods that were used to predetermine sample size OR if no sample-size calculation was performed, describe how sample sizes were chosen and provide a rationale for why these sample sizes are sufficient. For qualitative data, please indicate whether data saturation was considered, and what criteria were used to decide that no further sampling was needed.*
Data collection	*Provide details about the data collection procedure, including the instruments or devices used to record the data (e.g. pen and paper, computer, eye tracker, video or audio equipment) whether anyone was present besides the participant(s) and the researcher, and whether the researcher was blind to experimental condition and/or the study hypothesis during data collection.*
Timing	*Indicate the start and stop dates of data collection. If there is a gap between collection periods, state the dates for each sample cohort.*
Data exclusions	*If no data were excluded from the analyses, state so OR if data were excluded, provide the exact number of exclusions and the rationale behind them, indicating whether exclusion criteria were pre-established.*
Non-participation	*State how many participants dropped out/declined participation and the reason(s) given OR provide response rate OR state that no participants dropped out/declined participation.*
Randomization	*If participants were not allocated into experimental groups, state so OR describe how participants were allocated to groups, and if allocation was not random, describe how covariates were controlled.*

Ecological, evolutionary & environmental sciences study design

All studies must disclose on these points even when the disclosure is negative.

Study description	*Briefly describe the study. For quantitative data include treatment factors and interactions, design structure (e.g. factorial, nested, hierarchical), nature and number of experimental units and replicates.*
Research sample	*Describe the research sample (e.g. a group of tagged Passer domesticus, all Stenocereus thurberi within Organ Pipe Cactus National Monument), and provide a rationale for the sample choice. When relevant, describe the organism taxa, source, sex, age range and any manipulations. State what population the sample is meant to represent when applicable. For studies involving existing datasets, describe the data and its source.*
Sampling strategy	*Note the sampling procedure. Describe the statistical methods that were used to predetermine sample size OR if no sample-size calculation was performed, describe how sample sizes were chosen and provide a rationale for why these sample sizes are sufficient.*
Data collection	*Describe the data collection procedure, including who recorded the data and how.*
Timing and spatial scale	*Indicate the start and stop dates of data collection, noting the frequency and periodicity of sampling and providing a rationale for these choices. If there is a gap between collection periods, state the dates for each sample cohort. Specify the spatial scale from which the data are taken*
Data exclusions	*If no data were excluded from the analyses, state so OR if data were excluded, describe the exclusions and the rationale behind them, indicating whether exclusion criteria were pre-established.*
Reproducibility	*Describe the measures taken to verify the reproducibility of experimental findings. For each experiment, note whether any attempts to repeat the experiment failed OR state that all attempts to repeat the experiment were successful.*

Randomization	*Describe how samples/organisms/participants were allocated into groups. If allocation was not random, describe how covariates were controlled. If this is not relevant to your study, explain why.*
Blinding	*Describe the extent of blinding used during data acquisition and analysis. If blinding was not possible, describe why OR explain why blinding was not relevant to your study.*

Did the study involve field work? ☐ Yes ☒ No

Field work, collection and transport

Field conditions	*Describe the study conditions for field work, providing relevant parameters (e.g. temperature, rainfall).*
Location	*State the location of the sampling or experiment, providing relevant parameters (e.g. latitude and longitude, elevation, water depth).*
Access & import/export	*Describe the efforts you have made to access habitats and to collect and import/export your samples in a responsible manner and in compliance with local, national and international laws, noting any permits that were obtained (give the name of the issuing authority, the date of issue, and any identifying information).*
Disturbance	*Describe any disturbance caused by the study and how it was minimized.*

Reporting for specific materials, systems and methods

We require information from authors about some types of materials, experimental systems and methods used in many studies. Here, indicate whether each material, system or method listed is relevant to your study. If you are not sure if a list item applies to your research, read the appropriate section before selecting a response.

Materials & experimental systems

n/a	Involved in the study	
☐	☒	Antibodies
☐	☒	Eukaryotic cell lines
☒	☐	Palaeontology and archaeology
☐	☒	Animals and other organisms
☒	☐	Clinical data
☒	☐	Dual use research of concern
☒	☐	Plants

Methods

n/a	Involved in the study	
☒	☐	ChIP-seq
☐	☒	Flow cytometry
☒	☐	MRI-based neuroimaging

Antibodies

Antibodies used	Antibodies and other reagents used for cell staining are listed in Supplementary Table 2. Protocols for their use can be found in the respective methods section.<br>Used antibodies:<br>anti-V5-tag (Bio-Rad,MCA1360, clone: SV5-Pk1, lot#: 150547, 148239, dilutions: IF: 1:500; FACS: 3.1 µg/ml; MACS: 1 µg/ml; IHC(P):1:500; WB: 1:2,000); anti-β-actin-Dylight CW680(Bio-Rad, discontinued, clone: AbD12141, lot#: 0114, dilution: 1:5,000); anti-mouse IgG-IRDye 800CW (LI-COR, 926-32210, lot#: C91210-09, dilution: 1:20,000); anti-mouse IgG [F(ab')2]-AF488 (Invitrogen, A21204, lot#: 2155587, dilution: 1:20,000); anti-human EGFR(t)-PE (BioLegend, 352904, clone: AY13, lot#: B336514, dilution: 1:2,000); anti-V5-tag-PE (Life Technologies, 12-6796-42, clone: TCM5, lot#: 2301156, dilution: 1:500); anti-human CD3-APC (Life Technologies, 17-0038-42, clone: UCHT1, lot#: 2376138, dilution: 1:200); anti-human CD8-APC-efluor780 (eBioscience, 47-0086-42, clone: OKT8, lot#: 2611767, dilution: 1:100); anti-human CD45-krome-orange (Beckman Coulter, B36294, clone: J33, lot#: 200097, dilution: 1:50); Streptavidin-efluor450 (Life Technologies, 48-4317-82, lot#: 2527387, dilution: 1:50); Streptavidin-FITC (Biolegend, 405201, lot#: B309228, dilution: 1:400); anti-CD8-PE (eBioscience, 12-0086-42, clone: OKT8, lot#: 2504398, dilution: 1:50); anti-CD8-APC (BioLegend,301049, clone: RPA-T8, lot#: B368721, dilution: 1:200); anti-CD8-APC.eflour780 (eBioscience, 47-0086-42, clone: OKT8, lot#: 2611767, dilution: 1:100); anti-CD8- pacific orange ~ KO (Life Technologies, MHCD0830, clone: 3B5, lot#: 2375611, dilution: 1:50); anti-CD8-efluor450 (eBioscience, 48-0086-42, clone: OKT8, lot#: 2410932, dilution: 1:100); anti-CD69-APC (BioLegend, 310910, clone: FN50, lot#: B337763, dilution: 1:50); anti-CD3-AF488 (BioLegend, 300319, clone: HIT3a, lot#: B278329, dilution: 1:20); anti-CD4-AF488 (BioLegend, 317419, clone: OKT4, lot#: B292040, dilution: 1:20); anti-CXCR3 / CD183-AF488 (BioLegend, 353709, clone: G025H7, lot#: B264198, dilution: 1:20); anti-human CD19 (Cell Signaling Technology, 90176S , clone: D4V4B, lot#: 1, dilution: 1:600)
Validation	The DTPA-R reporter gene comprises the V5-tag, which can be bound by the anti-V5-tag antibody SV5-Pk1. The ability of this antibody to detect the DTPA-R reporter protein and DTPA-R labeled cells has been demonstrated for various techniques, including immune fluorescence (Fig. 1e), IHC(P) (Fig. 4j), MACS (Fig. 1h), and FACS (Fig. 1f).<br>The FACS panel for CAR-T cell characterization as well as the anti-CD19 antibody for IHC(P) have been used in previous studies and were validated with appropriate samples and controls.<br>The following antibodies were tested by the vendors.

Vendors tested for Flow cytometry, Immunofluorescence, Western Blot, Immunohistochemistry: anti-V5-tag (Bio-Rad)
Vendors tested for Western Blot: anti-β-actin (Bio-Rad) and anti-mouse IgG (LI-COR)
Vendors tested for Immunofluorescence: anti-mouse IgG [F(ab')2] (Invitrogen)
Vendors tested for Flow cytometry: anti-human EGFR(t) (BioLegend), anti-V5-tag (Life Technologies), anti-human CD3 (Life Technologies), anti-human CD8 (eBioscience), Streptavidin (Life Technologies), Streptavidin (Biolegend), anti-CD8 (eBioscience), anti-human CD45 (Beckman Coulter), anti-CD8 (BioLegend), anti-CD8 (eBioscience), anti-CD8 (Life Technologies), anti-CD8 (eBioscience), anti-CD69 (BioLegend), anti-CD3 (BioLegend), anti-CD4 (BioLegend), anti-CXCR3 / CD183 (BioLegend)
Vendors tested for Immunohistochemistry: anti-human CD19 (Cell Signaling Technology)

Eukaryotic cell lines

Policy information about cell lines and Sex and Gender in Research

Cell line source(s)	Jurkat T cell line obtained from Prof. Bernhard Küster, TU Munich (American Type Culture collection (ATCC), Manassas, VA; TIB-152), identity confirmed by Multiplex human Cell line Authentication Test on 20.08.2023).<br>Prostate carcinoma cell line PC3 (ATCC, catalogue number: CRL-1435).<br>Raji-GFP-fLuc cells expressing green fluorescent protein (GFP) and firefly luciferase (fLuc); obtained from Prof. Stanley Riddell, Fred Hutchinson Cancer Center Seattle, ATCC: CCL-86 transduced with GFP-fLuc (Hudecek, M. et al. 2015) identity confirmed by Multiplex human Cell line Authentication Test on 20.08.2023.<br>NALM6-GFP-fLuc cells obtained from Prof. Stanley Riddell, Fred Hutchinson Cancer Center Seattle, NALM6 ATCC: RL-3273 transduced with GFP-flLuc.<br>Human embryonic kidney (HEK293T) cells obtained from Prof. Gil Westmeyer, TU Munich (Sigma-Aldrich: ECACC 12022001) identity confirmed by Multiplex human Cell line Authentication Test on 20.08.2023.<br>HEK293CD19 cells (obtained from Prof. Stanley Riddell, ATCC_CRL-1573 transduced with CD19 identity confirmed by Multiplex human Cell line Authentication Test on 20.08.2023.
Authentication	Cell lines obtained from academic sources were authenticated by SNP profiling (Multiplexion, Heidelberg, Germany). The SNP profiles matched known profiles.
Mycoplasma contamination	Eucaryotic cells were regularly tested by PCR and found to be free of mycoplasma contamination.
Commonly misidentified lines<br>(See ICLAC register)	The cell lines used were compared to the Register of Misidentified Cell Lines (ver. 12).<br>Only HEK is considered a misidentified cell line. The HEK293T and HEK293-CD19 cell lines have been authenticated by SNP profiling, and the profiles obtained matched.

Palaeontology and Archaeology

Specimen provenance	*Provide provenance information for specimens and describe permits that were obtained for the work (including the name of the issuing authority, the date of issue, and any identifying information). Permits should encompass collection and, where applicable, export.*
Specimen deposition	*Indicate where the specimens have been deposited to permit free access by other researchers.*
Dating methods	*If new dates are provided, describe how they were obtained (e.g. collection, storage, sample pretreatment and measurement), where they were obtained (i.e. lab name), the calibration program and the protocol for quality assurance OR state that no new dates are provided.*

☐ Tick this box to confirm that the raw and calibrated dates are available in the paper or in Supplementary Information.

Ethics oversight	*Identify the organization(s) that approved or provided guidance on the study protocol, OR state that no ethical approval or guidance was required and explain why not.*

Note that full information on the approval of the study protocol must also be provided in the manuscript.

Animals and other research organisms

Policy information about studies involving animals; ARRIVE guidelines recommended for reporting animal research, and Sex and Gender in Research

Laboratory animals	Mice were purchased from Charles River Laboratories and used for experiments: C57BL/6 (C57BL/6NCrl, strain code 027), CD1-nude (Crl:CD1-Foxn1nu, strain code 086), and NSG (NOD.Cg-PrkdcSCIDIl2rgtm1Wjl/SzJ, strain code 614). Mice were 6 to 10 weeks old with a body weight of 15 to 25 g at the start of the experiments. Animals were kept at 45-60% humidity and 20-24°C.
Wild animals	The study did not involve wild animals.
Reporting on sex	Female animals were used for most experiments to decrease biological variation, except for the biodistribution study investigating the sex-specificity of [18F]F-DTPA biodistribution.
Field-collected samples	The study did not involve samples that were collected in the field.

Ethics oversight	Animal experiments were conducted in accordance with animal welfare regulations in Germany and with permission from the District Government of Upper Bavaria (approvals ROB-55.2-2532.Vet_216-15, Vet_21-127, Vet_02-21-41 and ROB-55.2-2532_Vet_02-18-162). The animal protocol was reviewed by the commission defined by §15 of the German animal protection act and received approval.

Note that full information on the approval of the study protocol must also be provided in the manuscript.

Clinical data

Policy information about clinical studies

All manuscripts should comply with the ICMJE guidelines for publication of clinical research and a completed CONSORT checklist must be included with all submissions.

Clinical trial registration	*Provide the trial registration number from ClinicalTrials.gov or an equivalent agency.*
Study protocol	*Note where the full trial protocol can be accessed OR if not available, explain why.*
Data collection	*Describe the settings and locales of data collection, noting the time periods of recruitment and data collection.*
Outcomes	*Describe how you pre-defined primary and secondary outcome measures and how you assessed these measures.*

Dual use research of concern

Policy information about dual use research of concern

Hazards

Could the accidental, deliberate or reckless misuse of agents or technologies generated in the work, or the application of information presented in the manuscript, pose a threat to:

No	Yes	
☐	☐	Public health
☐	☐	National security
☐	☐	Crops and/or livestock
☐	☐	Ecosystems
☐	☐	Any other significant area

Experiments of concern

Does the work involve any of these experiments of concern:

No	Yes	
☐	☐	Demonstrate how to render a vaccine ineffective
☐	☐	Confer resistance to therapeutically useful antibiotics or antiviral agents
☐	☐	Enhance the virulence of a pathogen or render a nonpathogen virulent
☐	☐	Increase transmissibility of a pathogen
☐	☐	Alter the host range of a pathogen
☐	☐	Enable evasion of diagnostic/detection modalities
☐	☐	Enable the weaponization of a biological agent or toxin
☐	☐	Any other potentially harmful combination of experiments and agents

Plants

Seed stocks	*Report on the source of all seed stocks or other plant material used. If applicable, state the seed stock centre and catalogue number. If plant specimens were collected from the field, describe the collection location, date and sampling procedures.*
Novel plant genotypes	*Describe the methods by which all novel plant genotypes were produced. This includes those generated by transgenic approaches, gene editing, chemical/radiation-based mutagenesis and hybridization. For transgenic lines, describe the transformation method, the number of independent lines analyzed and the generation upon which experiments were performed. For gene-edited lines, describe the editor used, the endogenous sequence targeted for editing, the targeting guide RNA sequence (if applicable) and how the editor was applied.*
Authentication	*Describe any authentication procedures for each seed stock used or novel genotype generated. Describe any experiments used to*

Authentication	*assess the effect of a mutation and, where applicable, how potential secondary effects (e.g. second site T-DNA insertions, mosiacism, off-target gene editing) were examined.*

ChIP-seq

Data deposition

☐ Confirm that both raw and final processed data have been deposited in a public database such as GEO.

☐ Confirm that you have deposited or provided access to graph files (e.g. BED files) for the called peaks.

Data access links<br>*May remain private before publication.*	*For "Initial submission" or "Revised version" documents, provide reviewer access links. For your "Final submission" document, provide a link to the deposited data.*
Files in database submission	*Provide a list of all files available in the database submission.*
Genome browser session<br>(e.g. UCSC)	*Provide a link to an anonymized genome browser session for "Initial submission" and "Revised version" documents only, to enable peer review. Write "no longer applicable" for "Final submission" documents.*

Methodology

Replicates	*Describe the experimental replicates, specifying number, type and replicate agreement.*
Sequencing depth	*Describe the sequencing depth for each experiment, providing the total number of reads, uniquely mapped reads, length of reads and whether they were paired- or single-end.*
Antibodies	*Describe the antibodies used for the ChIP-seq experiments; as applicable, provide supplier name, catalog number, clone name, and lot number.*
Peak calling parameters	*Specify the command line program and parameters used for read mapping and peak calling, including the ChIP, control and index files used.*
Data quality	*Describe the methods used to ensure data quality in full detail, including how many peaks are at FDR 5% and above 5-fold enrichment.*
Software	*Describe the software used to collect and analyze the ChIP-seq data. For custom code that has been deposited into a community repository, provide accession details.*

Flow Cytometry

Plots

Confirm that:

☒ The axis labels state the marker and fluorochrome used (e.g. CD4-FITC).

☒ The axis scales are clearly visible. Include numbers along axes only for bottom left plot of group (a 'group' is an analysis of identical markers).

☒ All plots are contour plots with outliers or pseudocolor plots.

☒ A numerical value for number of cells or percentage (with statistics) is provided.

Methodology

Sample preparation	Samples were either obtained from cell culture experiments (activation or proliferation assays, MACS) or collected from animal tissue or blood. Antibodies and other reagents used for cell staining are listed in Supplementary Table 2.
Instrument	LSR-Fortessa (Becton Dickinson) or CytoFLEX S (Beckman Coulter)
Software	FlowJo software (ver. 10.8.0 & 10.8.1; Becton Dickinson)
Cell population abundance	The exemplary gating strategy for CD8-positive CAR-T cells depicted in Extended Data Fig. 8c indicates the following cell abundance after gating.<br>0.819*0.989*0.858*0.627*0.648*0.999*0.352=0.099<br>The abundance of the exemplary $CD8^+$ CAR-T cells is ~10% which can be reliably detected and quantified.

Gating strategy

A representative gating strategy for CD8-positive CAR-T cells starting from samples collected from animal studies can be seen in Extended Data Fig. 8c.
In short, 1) the target cell population and counting beads were identified in a SSC-H/FSC-H plot, 2) doublets were excluded using a SSC-H/FSC-W plot, 3) dead cells that were positively stained by propidium iodide were excluded (live cell gating), 4) huCD45-pos cells were gated (leukocyte gating), 5) endogenous GFP expression in Raji tumor cells and CD3 staining of T cells was used to identify respective cell populations, 6) Streptavidin (binding to the Strep-tag within the CAR) and antibodies against the V5-tag or the EGFRt allowed the characterization of the CAR-T population, finally 7) CD8 antibody stain allowed the differentiation between CD4 and CD8 T cells.

☒ Tick this box to confirm that a figure exemplifying the gating strategy is provided in the Supplementary Information.

Magnetic resonance imaging

Experimental design

Design type	*Indicate task or resting state; event-related or block design.*
Design specifications	*Specify the number of blocks, trials or experimental units per session and/or subject, and specify the length of each trial or block (if trials are blocked) and interval between trials.*
Behavioral performance measures	*State number and/or type of variables recorded (e.g. correct button press, response time) and what statistics were used to establish that the subjects were performing the task as expected (e.g. mean, range, and/or standard deviation across subjects).*

Acquisition

Imaging type(s)	*Specify: functional, structural, diffusion, perfusion.*
Field strength	*Specify in Tesla*
Sequence & imaging parameters	*Specify the pulse sequence type (gradient echo, spin echo, etc.), imaging type (EPI, spiral, etc.), field of view, matrix size, slice thickness, orientation and TE/TR/flip angle.*
Area of acquisition	*State whether a whole brain scan was used OR define the area of acquisition, describing how the region was determined.*

Diffusion MRI ☐ Used ☐ Not used

Preprocessing

Preprocessing software	*Provide detail on software version and revision number and on specific parameters (model/functions, brain extraction, segmentation, smoothing kernel size, etc.).*
Normalization	*If data were normalized/standardized, describe the approach(es): specify linear or non-linear and define image types used for transformation OR indicate that data were not normalized and explain rationale for lack of normalization.*
Normalization template	*Describe the template used for normalization/transformation, specifying subject space or group standardized space (e.g. original Talairach, MNI305, ICBM152) OR indicate that the data were not normalized.*
Noise and artifact removal	*Describe your procedure(s) for artifact and structured noise removal, specifying motion parameters, tissue signals and physiological signals (heart rate, respiration).*
Volume censoring	*Define your software and/or method and criteria for volume censoring, and state the extent of such censoring.*

Statistical modeling & inference

Model type and settings	*Specify type (mass univariate, multivariate, RSA, predictive, etc.) and describe essential details of the model at the first and second levels (e.g. fixed, random or mixed effects; drift or auto-correlation).*
Effect(s) tested	*Define precise effect in terms of the task or stimulus conditions instead of psychological concepts and indicate whether ANOVA or factorial designs were used.*

Specify type of analysis: ☐ Whole brain ☐ ROI-based ☐ Both

Statistic type for inference (See Eklund et al. 2016)	*Specify voxel-wise or cluster-wise and report all relevant parameters for cluster-wise methods.*
Correction	*Describe the type of correction and how it is obtained for multiple comparisons (e.g. FWE, FDR, permutation or Monte Carlo).*

Models & analysis

n/a	Involved in the study
☐	☐ Functional and/or effective connectivity
☐	☐ Graph analysis
☐	☐ Multivariate modeling or predictive analysis

Functional and/or effective connectivity

Report the measures of dependence used and the model details (e.g. Pearson correlation, partial correlation, mutual information).

Graph analysis

Report the dependent variable and connectivity measure, specifying weighted graph or binarized graph, subject- or group-level, and the global and/or node summaries used (e.g. clustering coefficient, efficiency, etc.).

Multivariate modeling and predictive analysis

Specify independent variables, features extraction and dimension reduction, model, training and evaluation metrics.
